# A Mizuno-Todd-Ye predictor-corrector infeasible-interior-point method for symmetric optimization with the arc-search strategy

**DOI:** 10.1186/s13660-017-1565-y

**Published:** 2017-11-21

**Authors:** Ximei Yang, Yinkui Zhang

**Affiliations:** Henan Engineering Laboratory for Big Data Statistical Analysis and Optimal Control, School of Mathematics and Information Science, Henan Normal University, East of Construction Road, Xinxiang, 453007 P.R. China

**Keywords:** 90C05, 90C25, 90C51, symmetric optimization, infeasible-interior-point method, arc-search, Mizuno-Todd-Ye predictor-corrector, iteration complexity

## Abstract

In this paper, we propose a Mizuno-Todd-Ye predictor-corrector infeasible-interior-point method for symmetric optimization using the arc-search strategy. The proposed algorithm searches for optimizers along the ellipses that approximate the central path and ensures that the duality gap and the infeasibility have the same rate of decline. By analyzing, we obtain the iteration complexity $\mathcal{O}(r\log \varepsilon^{-1})$ for the Nesterov-Todd direction, where *r* is the rank of the associated Euclidean Jordan algebra and *ε* is the required precision. To our knowledge, the obtained complexity bounds coincide with the currently best known theoretical complexity bounds for infeasible symmetric optimization.

## Introduction

The purpose of this paper is to propose a Mizuno-Todd-Ye predictor-corrector (MTY-PC) infeasible-interior-point method (infeasible-IPM) for symmetric optimization (SO) by using Euclidean Jordan algebra (EJA). Recently, SO has caused widespread concern, because it provides a unified framework for various convex optimizations including linear optimization (LO), second-order cone optimization (SOCO), and semi-definite optimization (SDO) as special cases. Meanwhile, there are many methods for solving SO. Particularly, the interior-point method (IPM), which was first proposed by Karmarkar [1], is an important kind of classification algorithm. There is extensive literature on the analysis of IPMs for SO [2–11].

Nowadays, it is broadly accepted that the primal-dual IPM is the most efficient IPM and includes the Mehrotra predictor-corrector (M-PC) algorithm [12] and the MTY-PC algorithm [13] as two typical representatives. For literature on the research of the M-PC algorithm, please see [7, 8, 14]. In the nineties of the last century, researchers began to focus on the MTY-PC algorithm [15–22], because it had the property of the best iteration complexity obtained so far for all the IPMs. Later, some researchers further studied other aspects of the MTY-PC algorithm [23–25]. Recently, Kitahara [26] proposed a simple variant of the MTY-PC algorithm for LO, and Yang [27] extended the MTY-PC algorithm to SO. Inspired by their works, we present an $\mathcal{O}(r\log\varepsilon ^{-1})$-iteration complexity MTY-PC algorithm for SO. Moreover, the proposed algorithm will use the infeasible starting, which is found to be easy in practice. This kind of IPM is called infeasible-IPM and is studied in the literature [6, 7, 9, 22, 28–31].

Moreover, the proposed algorithm in this paper has another invention, i.e., the arc-search strategy. Yang [32–34] first developed the arc-search algorithm that searches for optimizers along an ellipse that is an approximation of the central path and gave some of the advantages of the arc-search algorithm. In order to further study the advantages of the arc-search algorithm, Yang [35, 36] proposed two infeasible-IPMs for LO and SO, and respectively obtained the $\mathcal{O}(n^{5/4}\log\varepsilon^{-1})$-iteration complexity for LO and the $\mathcal{O}(r^{5/4}\log\varepsilon^{-1})$ and $\mathcal {O}(r^{7/4}\log\varepsilon^{-1})$-iteration complexity, where *n* is the larger dimension of a standard LO, *r* is the rank of the associated EJA and *ε* is the required precision. In order to improve the iteration complexity of infeasible-IPM, we will add the arc-search strategy to the MTY-PC algorithm.

In this paper, we propose an MTY-PC infeasible-IPM for SO. The proposed algorithm uses the arc-search strategy and ensures that the duality gap and the infeasibility have the same rate of decline. By analyzing, we achieve the $\mathcal{O}(r\log\varepsilon^{-1})$ iteration complexity for the Nesterov-Todd (NT) direction. To our knowledge, this is the best iteration complexity obtained so far for an infeasible SO problem.

The outline of this paper is organized as follows. In Section 2, we briefly introduce some key results on EJA. In Section 3, we give some preliminary discussions for an algorithm and propose the algorithm. In Section 4, we establish the iteration complexity for the proposed algorithm. Finally, we close the paper by some conclusions.

## Euclidean Jordan algebra

In order to ensure the integrity of this paper, we give some results for EJA. Most of these can be found in [5, 37].

EJA is a triple $(\mathcal{J},\circ,\langle\cdot,\cdot\rangle)$, where $(\mathcal{J},\langle\cdot,\cdot\rangle)$ is an *n*-dimensional inner product space over $\mathbb{R}$ and $(x,y)\mapsto x \circ y:\mathcal{J}\times\mathcal{J}\mapsto\mathcal{J}$ is a bilinear mapping satisfying the following conditions:

- $x\circ y=y\circ x$ for all $x,y\in\mathcal{J}$.
- $x\circ(x^{2}\circ y)=x^{2}\circ(x\circ y)$ for all $x,y\in\mathcal{J}$, where $x^{2}:=x\circ x$.
- $\langle x\circ y,z\rangle=\langle y ,x\circ z \rangle$ for all $x,y,z\in\mathcal{J}$.

We call $x\circ y$ the Jordan product of *x* and *y* and define the inner product as $\langle x,y\rangle:=\operatorname{tr}(x\circ y)$. If there exists an element *e* such that $x\circ e=e\circ x=x$ for all $x\in\mathcal{J}$, then *e* is called the multiplicative identity element of EJA. For any $x\in\mathcal{J}$, the degree of *x* is denoted by $\operatorname{deg}(x)$, which is defined as the smallest integer *k* such that the set $\{e,x,x^{2},\ldots,x^{k}\}$ is linearly dependent. The rank of $\mathcal{J}$, simply denoted by *r*, is the maximum of $\operatorname{deg}(x)$ for all $x\in\mathcal{J}$. For EJA $\mathcal{J}$, the corresponding cone of squares $\mathcal{K}:=\{x^{2}:x\in\mathcal{J}\}$ is indeed a symmetric cone. A cone is symmetric if and only if it is the cone of squares of some EJA. Moreover, $\operatorname{int} \mathcal{K}$ denotes the interior of the symmetric cone $\mathcal{K}$.

An idempotent *c* is a nonzero element of $\mathcal{J}$ such that $c^{2}=c$. An idempotent is primitive if it cannot be written as the sum of two idempotents. Two idempotents $c_{1}$ and $c_{2}$ are orthogonal if $c_{1}\circ c_{2} = 0$. A complete system of orthogonal idempotents is a set $\{c_{1},\ldots,c_{k}\}$ of idempotents, where $c_{i}\circ c_{j}=0$ for all $i\neq j$, and $c_{1}+\cdots+c_{k}=e$. A complete system of orthogonal primitive idempotents is called a Jordan frame.

### Theorem 2.1

(Spectral decomposition [37, Theorem III.1.2])

*Let* $\mathcal{J}$ *be EJA with rank* *r*. *Then*, *for every* $x\in\mathcal{J}$, *there exist a Jordan frame* $\{c_{1},\ldots,c_{r}\}$ *and real numbers* $\lambda_{1},\ldots,\lambda_{r}$ *such that*

$$x=\lambda_{1}c_{1}+\lambda_{2}c_{2}+ \cdots+\lambda_{r}c_{r}=\sum_{i=1}^{r} \lambda_{i}c_{i}, $$

*where the* $\lambda_{i}$ *’s are called the eigenvalues of* *x*.

Let $x=\sum_{i=1}^{r}\lambda_{i}c_{i}$ be the spectral decomposition of *x*. We say $x\in\mathcal{K}$ if and only if $\lambda_{i}\geq0$ and $x\in\operatorname{int} \mathcal{K}$ if and only if $\lambda_{i}>0$ for all $i=1,\ldots,r$. Define the square root $x^{1/2}:=\sum\lambda _{i}^{1/2}c_{i}$ for $x\in\mathcal{K}$, the inverse $x^{-1}:=\sum\lambda_{i}^{-1}c_{i}$, $\forall\lambda_{i}\neq0$ and the trace $\operatorname{tr}(x):=\sum\lambda _{i}$ for $x\in\mathcal{J}$ and the determinant $\det(x):=\prod\lambda _{i}$ for $x\in\mathcal{J}$. We also define the spectral norm $\|x\| _{2}:=\max_{i}|\lambda_{i}|$ and the Frobenius norm $\|x\|_{F}:=\sqrt{\langle x,x\rangle}=\sqrt{\sum\lambda^{2}_{i}}$.

Since ‘∘’ is bilinear for every $x\in\mathcal{J}$, there exists a linear operator $L_{x}$ such that, for every $y\in\mathcal{J}$, $x\circ y=L_{x}y$. In particular, $L_{x}e = x$ and $L_{x}x=x^{2}$. We say that two elements $x, y\in\mathcal{J}$ operator commute if $L_{x}L_{y}=L_{y}L_{x}$. It can be proven that *x* and *s* operator commute if and only if they share a common Jordan frame [5, Theorem 27]. For each $x,y\in\mathcal{J}$, define $Q_{x,y}:=L_{x}L_{y}+L_{y}L_{x}-L_{x\circ y}$, $Q_{x}:=Q_{x,x}=2L^{2}_{x}-L_{x^{2}}$ and $Q_{x}$ is called the quadratic representation of *x*. The following is a useful proposition of quadratic representation.

### Proposition 2.2

([5, Proposition 21])

*Let* *x*, *y*, $p\in\operatorname{int} \mathcal{K}$ *and define* $\tilde{x}:=Q_{p}x$ *and* $\tilde{y}:=Q_{p^{-1}}y$, *then* $Q_{x^{1/2}}y$, $Q_{y^{1/2}}x$ *and* $Q_{\tilde{x}^{1/2}}\tilde{y}$ *have the same spectrum*.

## Preliminary discussions and algorithm

### SO problem and ellipse approximate center

First, we give the standard form of SO and its dual form, as follows:

1 $$\begin{aligned}& \text{(P)}\quad \min\langle c,x\rangle, \quad\text{s.t.}\quad Ax=b,\quad x\in\mathcal{K}, \end{aligned}$$

2 $$\begin{aligned}& \text{(D)}\quad \max\langle b,y\rangle,\quad \text{s.t.}\quad A^{*}y+s=c,\quad s\in\mathcal{K},y\in {\mathbb{R}^{m},} \end{aligned}$$

where $c\in\mathcal{J}$, $b\in{\mathbb{R}^{m}}$, *A* is a linear operator that maps $\mathcal{J}$ into ${\mathbb{R}^{m}}$ and $A^{*}$ is its adjoint operator such that $\langle x,A^{*}y\rangle=\langle Ax,y\rangle$ for all $x\in\mathcal{J}$, $y\in{\mathbb{R}^{m}}$.

Moreover, we denote the sets of optimal solutions of (P) and (D) by $\mathcal{P}^{*}$ and $\mathcal{D}^{*}$, and assume that *A* is surjective and $\mathcal{F}^{0}\neq\emptyset$, where $\mathcal{F}^{0}$ indicates a primal-dual strict feasibility set that is defined by

$$\mathcal{F}^{0}:=\bigl\{ (x,y,s)\in\operatorname{int }\mathcal{K} \times{\mathbb {R}^{m}}\times\operatorname{int }\mathcal{K}: Ax=b, A^{*}y+s=c\bigr\} . $$

The Karush-Kuhn-Tucker (KKT) conditions for (P) and (D) are given by

3 $$ Ax=b, \quad x\in\mathcal{K},\qquad A^{*}y+s=c,\quad s\in\mathcal{K},y\in{ \mathbb{R}^{m}},\qquad x\circ s=0, $$

where $x\circ s=0$ is called the complementarity slackness condition.

By relaxing $x\circ s=0$ with $x\circ s=\mu e$, we obtain

4 $$ Ax=b,\quad x\in\mathcal{K},\qquad A^{*}y+s=c,\quad s\in\mathcal{K},y\in{ \mathbb{R}^{m}},\qquad x\circ s =\mu e, $$

where $\mu=\langle x, s\rangle/r>0$ is called the duality gap.

System (4) has unique solutions $(x(\mu),y(\mu),s(\mu))$, the set of which is called the central path, which is denoted by

5 $$ \mathcal{C}=\bigl\{ \bigl(x(\mu),y(\mu),s(\mu)\bigr):\mu>0\bigr\} . $$

In this paper, we will use the idea of Yang [32–34], which is that the central path $\mathcal{C}$ is replaced by an ellipse Ω, where Ω is defined as follows:

6 $$ \Omega= \bigl\{ \bigl(x(\theta),y(\theta),s(\theta)\bigr):\bigl(x( \theta),y(\theta ),s(\theta)\bigr)=\mathbf{a}\cos(\theta)+\mathbf{b}\sin(\theta)+ \mathbf {c} \bigr\} , $$

where $\mathbf{a}\in {\mathbb{R}^{2n+m}}$ and $\mathbf{b}\in {\mathbb {R}^{2n+m}}$ are the axes of the ellipse perpendicular to each other, and $\mathbf{c}\in {\mathbb{R}^{2n+m}}$ is the center of the ellipse.

For the point $z=(x,y,s)=(x(\theta_{0}),y(\theta_{0}),s(\theta_{0})) \in \Omega$, we require its first and second derivatives such that

7 $$\begin{aligned}& A \dot{x}={r}_{p}, \qquad A^{*} \dot{y}+ \dot{s}={r}_{d},\qquad s\circ \dot{x} +x \circ\dot{s}=x\circ s, \end{aligned}$$

8 $$\begin{aligned}& A \ddot{x}=0,\qquad A^{*}\ddot{y}+\ddot{s}=0,\qquad s\circ\ddot{x}+x\circ\ddot{s}=-2\dot{x} \dot{s}, \end{aligned}$$

where ${r}_{p}={A}{x}-b$ and ${r}_{d}=A^{*}y+{s}-{c}$.

Systems (7) and (8) do not always have a unique solution due to the fact that *x* and *s* do not operator commute in general. To overcome this difficulty, we apply a scaling scheme that follows from [5, Lemma 28]. For the scaling point $p\in\operatorname{int }\mathcal{K}$, there are several appropriate choices (see [38]). In this paper, we select the classical NT-scaling point that is

9 $$ p=\bigl[Q_{x^{1/2}}(Q_{x^{1/2}}s)^{-1/2} \bigr]^{-1/2}=\bigl[Q_{s^{-1/2}}(Q_{s^{1/2}}x)^{1/2} \bigr]^{-1/2}, $$

which was first proposed by Nesterov and Todd for self-scaled cones [4] and then adapted by Faybusovich [3] for symmetric cones.

### Foundation of the MTY-PC algorithm

Since the MTY-PC algorithm requires two matrix factorizations and at most three back-solves for each iteration, it is generally divided into two steps, which are the predictor step and the corrector step.

In the predictor step, using *p* in (9), systems (7) and (8) are rewritten as

10 $$\begin{aligned}& \tilde{A} \dot{\tilde{x}}=\tilde{r}_{p},\qquad \tilde{A}^{*} \dot{y}+ \dot{\tilde{s}}=\tilde{r}_{d},\qquad \tilde{s}\circ\dot{\tilde{x}}+ \tilde{x}\circ\dot{\tilde{s}}=\tilde {x}\circ\tilde{s}, \end{aligned}$$

11 $$\begin{aligned}& \tilde{A} \ddot{\tilde{x}}=0, \qquad \tilde{A}^{*}\ddot{ y}+ \ddot{ \tilde{s}}=0, \qquad\tilde{s}\circ\ddot{\tilde{x}}+\tilde{x}\circ\ddot{\tilde{s}}=-2\dot {\tilde{x}}\circ\dot{\tilde{s}}, \end{aligned}$$

where $\tilde{A}=AQ_{p^{-1}}$, $\tilde{c}=Q_{p^{-1}}c$, $\tilde{x}=Q_{p}x$, $Q_{p^{-1}}s=\tilde{s}$ and $\dot{\tilde{x}}=Q_{p} \dot {x}$, $\dot{\tilde{s}}=Q_{p^{-1}}\dot{s}$, $\ddot{\tilde{x}}=Q_{p} \ddot {x}$, $\ddot{\tilde{s}}=Q_{p^{-1}} \ddot{s}$, $\tilde{r}_{p}=\tilde{A}\tilde{x}-b$, $\tilde{r}_{d}=\tilde{A}^{*}y+\tilde {s}-\tilde{c}$.

By solving systems (10) and (11), we obtain the predictor directions $( \dot{\tilde{x}}, \dot{\tilde{y}}, \dot{\tilde{s}})$ and $(\ddot{\tilde {x}},\ddot{\tilde{y}},\ddot{\tilde{s}})$ and have the following lemma.

#### Lemma 3.1

([34, Theorem 3.1])

*Let* $(\tilde{x}(\theta ),y(\theta),\tilde{s}(\theta))$ *be an arc defined by* (6) *passing through a point* $(\tilde{x},y, \tilde {s})$, *and its first and second derivatives at* $(\tilde{x},y, \tilde {s})$ *be* $( \dot{\tilde{x}}, \dot{\tilde{y}}, \dot{\tilde{s}})$ *and* $(\ddot{\tilde{x}},\ddot{\tilde{y}},\ddot{\tilde{s}})$, *which are defined by* (10) *and* (11). *Then an ellipsoidal approximation of the central path is given by*

12a $$\begin{aligned}& \tilde{x}(\theta):=\tilde{x}-\sin(\theta) \dot{\tilde{x}}+\bigl(1-\cos (\theta) \bigr)\ddot{\tilde{x}}, \end{aligned}$$

12b $$\begin{aligned}& \tilde{s}(\theta):=\tilde{s}-\sin(\theta)\dot{\tilde{s}}+\bigl(1-\cos(\theta ) \bigr) \ddot{\tilde{s}}, \end{aligned}$$

12c $$\begin{aligned}& y(\theta):=y-\sin(\theta) \dot{y}+\bigl(1-\cos(\theta)\bigr) \ddot{y}. \end{aligned}$$

Using (12a), (12b), (12c), the third equations in (10) and (11), we have

13 $$ \tilde{x}(\theta)\circ\tilde{s}(\theta)=\bigl(1-\sin(\theta)\bigr) \tilde{x}\circ \tilde{s}-g^{2}(\theta)\dot{\tilde{x}}\circ\dot{ \tilde{s}}+ g(\theta)\sin(\theta)\xi+g^{2}(\theta)\ddot{\tilde{x}}\circ \ddot{\tilde{s}}, $$

where $g(\theta)= (1-\cos(\theta) )$, $\xi=\dot{\tilde{x}}\circ \ddot{\tilde{s}}+\dot{\tilde{s}}\circ\ddot{\tilde{x}}$.

Furthermore, using (13), we have

$$\begin{aligned} \bigl\langle \tilde{x}(\theta), \tilde{s}(\theta)\bigr\rangle =\bigl(1- \sin(\theta)\bigr)\mu r-g^{2}(\theta)\operatorname{tr}(\dot{\tilde{x}} \circ\dot{\tilde {s}})+g(\theta)\sin(\theta)\operatorname{tr}(\xi). \end{aligned}$$

In what follows, we discuss a method for selecting the predictor step. Firstly, we give the neighborhood that is used in this paper as follows:

14 $$ \mathcal{N}_{F}(\gamma)=\bigl\{ (x,y,s)\in \operatorname{int }\mathcal{K}\times {\mathbb{R}^{m}}\times \operatorname{int }\mathcal{K}: \|w-\mu{e}\|_{F}\leq \gamma\mu\bigr\} , $$

where $w=Q_{x^{1/2}}s$, $0<\gamma<1$.

The neighborhood $\mathcal{N}_{F}(\gamma)$ has some important properties, which are given in the following proposition. For more details, readers are referred to [5].

#### Proposition 3.2

*Let* $\mathcal{N}_{F}(\gamma)$ *be defined in* (14) *and* $w=Q_{x^{1/2}}s$, *then*

- *The neighborhood* $\mathcal{N}_{F}(\gamma)$ *is scaling invariant*.
- $\|w-\mu{e}\|_{F}\leq\gamma\mu$ *implies* $\lambda_{\min }(w)\geq\beta\mu$, *where* $\beta=1-\gamma$.

Now, we give the method of selecting the predictor step, which is to find the largest positive $\bar{\theta}\in(0,\pi/2]$ and to satisfy for all $\theta\in(0,\bar{\theta}]$ that

15 $$\begin{aligned}& \big\| \tilde{x}(\theta)\circ\tilde{s}(\theta)-\bigl(1-\sin(\theta)\bigr)\mu e \big\| _{F} \leq2\gamma\bigl(1-\sin(\theta)\bigr)\mu, \end{aligned}$$

16 $$\begin{aligned}& \tilde{x}(\theta)\in\operatorname{int} \mathcal{K}, \qquad\tilde{s}(\theta )\in \operatorname{int} \mathcal{K}. \end{aligned}$$

In the corrector step, we define $(\bar{x},\bar{y},\bar{s})=(Q_{p^{-1}}\tilde{x}(\bar{\theta}),y(\bar {\theta}),Q_{p}\tilde{s}(\bar{\theta}))$ and calculate the corrector direction $(\triangle x,\triangle y,\triangle s )$ by

17 $$ {A}\triangle{x}=0,\qquad {A}^{*}\triangle y+\triangle{s}=0,\qquad \bar{s} \circ\triangle{x}+ \bar{x}\circ\triangle{s}= r_{c}, $$

where $r_{c}=(1-\sin(\bar{\theta}))\mu e-\bar{x}\circ\bar{s}$.

Similarly, system (17) does not always have a unique solution. Thus, we need to choose an NT-scaling point $p_{1}$ such that

$$p_{1}=\bigl[Q_{\bar{x}^{1/2}}(Q_{\bar{x}^{1/2}}\bar{s})^{-1/2} \bigr]^{-1/2}=\bigl[Q_{\bar {s}^{-1/2}}(Q_{\bar{s}^{1/2}}\bar{x})^{1/2} \bigr]^{-1/2}. $$

The scaling corrector direction is given by solving the following system:

18 $$ \hat{A}\triangle\hat{x}=0,\qquad \hat{A}^{*}\triangle y+\triangle \hat{s}=0,\qquad \hat{s}\circ\triangle\hat{x}+\hat{x}\circ\triangle\hat{s}= \hat{r}_{c}, $$

where $\hat{x}=Q_{p_{1}}\bar{x}$, $\hat{s}=Q_{p_{1}^{-1}}\bar{s}$, $\hat {A}=Q_{p_{1}^{-1}}A$, $\hat{r}_{c}=(1-\sin(\bar{\theta}))\mu e-\hat{x}\circ \hat{s}$.

Eventually, the next iteration point is updated by

19 $$ \bigl(\hat{x}(\bar{\theta}),y(\bar{\theta}),\hat{s}(\bar{\theta}) \bigr) :=(\hat{x},\hat{y},\hat{s})+(\triangle\hat{x},\triangle{y},\triangle \hat{s}). $$

In what follows, we give two useful expressions

20 $$\begin{aligned}& \begin{aligned}[b] \hat{x}(\bar{\theta})\circ\hat{s}(\bar{\theta})&=\hat{x}\circ\hat {s}+\bigl(1- \sin(\bar{\theta})\bigr)\mu e-\hat{x}\circ\hat{s}+\triangle\hat {x}\circ\triangle \hat{s} \\ &=\bigl(1-\sin(\bar{\theta})\bigr)\mu e+\triangle\hat{x}\circ\triangle\hat{s},\end{aligned} \end{aligned}$$

21 $$\begin{aligned}& \mu(\bar{\theta})=\frac{1}{r}\bigl\langle \hat{x}(\bar{\theta}),\hat{s}( \bar {\theta})\bigr\rangle = \bigl(1-\sin(\bar{\theta})\bigr)\mu. \end{aligned}$$

### Framework of the MTY-PC algorithm

Based on the previous analysis, we state the generic framework of the proposed MTY-PC algorithm in this paper.

#### Algorithm 1

Let $\varepsilon>0$, $\gamma\leq1/4$, $(x^{0},y^{0},s^{0})\in\mathcal{N}_{F}(\gamma)$ and $\mu^{0}=\langle x^{0},s^{0}\rangle/r$, $\phi^{0}=1$, $k:=0$.

Step 1: If $x^{k}\in\operatorname{int} \mathcal{K}$, $s^{k}\in \operatorname{int} \mathcal{K}$ and $\phi^{k}\leq\varepsilon$, then stop.
Step 2: (Predictor step) The predictor directions $(\dot{\tilde{x}}^{k},\dot{y}^{k},\dot{\tilde{s}}^{k})$, $(\ddot{\tilde {x}}^{k},\ddot{y}^{k}, \ddot{\tilde{s}}^{k})$ are obtained by solving the linear system (10), (11) and the largest positive $\bar{\theta}^{k}\in(0,\pi/2]$ is computed by solving (16) and (15). Set $(\bar{x}^{k},\bar{y}^{k},\bar{s}^{k})=(Q_{p^{-1}}\tilde{x}^{k+1}(\bar {\theta}^{k}), y^{k+1}(\bar{\theta}^{k}),Q_{p}\tilde{s}^{k+1}(\bar{\theta}^{k}))$.
Step 3: (Corrector step) Solve corrector direction $(\triangle{\hat{x}}^{k},\triangle{y}^{k},\triangle{\hat{s}}^{k})$ from (18). Let $(\hat{x}^{k+1},y^{k+1},\hat{s}^{k+1})=(\hat{x}^{k}(\bar{\theta }^{k}),y^{k}(\bar{\theta}^{k}),\hat{s}^{k}(\bar{\theta}^{k}))$ and $({x}^{k+1},y^{k+1},{s}^{k+1})=(Q_{p_{1}^{-1}}\hat{x}^{k+1}, y^{k+1}, Q_{p_{1}}\hat{s}^{k+1})$. Go to Step 4.
Step 4: Compute $\mu^{k+1}=\frac{\langle\hat{x}^{k}(\bar{\theta }^{k}),\hat{s}^{k}(\bar{\theta}^{k})\rangle}{r}=\frac{\langle x^{k+1},s^{k+1}\rangle}{r}$ and $\phi^{k+1}=(1-\sin(\bar{\theta}^{k}))\phi^{k}$. Set $k:=k+1$ and go to Step 1.

To analyze complexity, we give two remarks for Algorithm 1.

#### Remark 1

Let $\{(x^{k},y^{k},s^{k})\}$ be generated by Algorithm 1, and $\phi^{k+1}\in[0,1]$ such that $\phi^{k+1}=\prod^{k}_{i=0}(1-\sin(\bar{\theta}^{i}))$. Then $r_{p}^{k+1}= Ax^{k+1}-b=\phi^{k+1}r_{p}^{0}$, $r_{d}^{k+1}=A^{*}y^{k+1}+s^{k+1}-c=\phi^{k+1}r_{d}^{0}$, $\mu^{k+1}=\frac {\langle x^{k+1},s^{k+1}\rangle}{r}=\phi^{k+1}\mu^{0}$ for $k\geq0$.

#### Proof

Using (7), (8), (12a), (12b), (12c), (17), (18), by calculating directly, we have

$$\begin{aligned} r_{p}^{k+1}&=Ax^{k+1}-b=AQ_{p_{1}^{-1}} \hat{x}^{k+1}-b=AQ_{p_{1}^{-1}}\hat {x}^{k}\bigl(\bar{ \theta}^{k}\bigr)-b \\ &=AQ_{p_{1}^{-1}} \bigl(\hat{x}^{k}+\triangle\hat{x}^{k} \bigr)-b=A\bar {x}^{k}+A\triangle x^{k}-b \\ &=A Q_{p^{-1}}\tilde{x}^{k+1}\bigl(\bar{\theta}^{k} \bigr)-b \\ &=A Q_{p^{-1}} \bigl[\tilde{x}^{k}-\sin\bigl(\bar{ \theta}^{k}\bigr) \dot{\tilde {x}}^{k}+\bigl(1-\cos\bigl( \bar{\theta}^{k}\bigr)\bigr)\ddot{\tilde{x}}^{k} \bigr]-b \\ &=A\bigl[x^{k}-\sin\bigl(\bar{\theta}^{k}\bigr) \dot{x}^{k}+\bigl(1-\cos\bigl(\bar{\theta }^{k}\bigr)\bigr) \ddot{x}^{k}\bigr]-b \\ &=Ax^{k}-\sin\bigl(\bar{\theta}^{k}\bigr)A \dot{x}^{k}+\bigl(1-\cos\bigl(\bar{\theta }^{k}\bigr)\bigr)A \ddot{x}^{k}-b \\ &=\bigl[1-\sin\bigl(\bar{\theta}^{k}\bigr)\bigr]r_{p}^{k}= \phi^{k+1}r_{p}^{0}. \end{aligned}$$

In the same way, we have $r_{d}^{k+1}=[1-\sin(\bar{\theta }^{k})]r_{p}^{k}=\phi^{k+1}r_{d}^{0}$.

In what follows, we focus on proving the last inequality and have

$$\begin{aligned} \mu^{k+1}&=\frac{\langle x^{k+1},s^{k+1}\rangle}{r}=\frac{\langle Q_{p_{1}^{-1}}\hat{x}^{k+1},Q_{p_{1}}\hat{s}^{k+1}\rangle}{r}=\frac{\langle \hat{x}^{k+1}, \hat{s}^{k+1}\rangle}{r} \\ &=\frac{\langle\hat{x}^{k}(\bar{\theta}^{k}),\hat{s}^{k}(\bar{\theta }^{k})\rangle}{r} =\frac{\langle\hat{x}^{k}+\triangle\hat{x}^{k},\hat{s}^{k}+\triangle \hat{s}^{k}\rangle}{r} \\ &=\frac{1}{r} \bigl[\bigl\langle \hat{x}^{k} \circ \hat{s}^{k}, e\bigr\rangle +\bigl\langle \hat{x}^{k}\circ \triangle\hat{s}^{k}+\hat{s}^{k} \circ \triangle \hat{x}^{k}, e\bigr\rangle +\bigl\langle \triangle\hat{x}^{k} \circ \triangle\hat{s}^{k}, e\bigr\rangle \bigr] \\ &=\frac{1}{r} \bigl[\bigl\langle Q_{p_{1}^{-1}}\tilde{x}^{k} \bigl(\bar{\theta}^{k}\bigr) \circ Q_{p_{1}}\tilde{s}^{k} \bigl(\bar{\theta}^{k}\bigr), e\bigr\rangle +\bigl\langle \hat {r}^{k}_{c}, e\bigr\rangle \bigr] \\ &=\frac{1}{r} \bigl[\bigl\langle \tilde{x}^{k}\bigl(\bar{ \theta}^{k}\bigr) \circ\tilde {s}^{k}\bigl(\bar{ \theta}^{k}\bigr), e\bigr\rangle +\bigl\langle \bigl(1-\sin\bigl(\bar{ \theta}^{k}\bigr)\bigr)\mu^{k} e-\tilde{x}^{k} \bigl(\bar{\theta}^{k}\bigr)\circ\tilde{s}^{k}\bigl(\bar{ \theta}^{k}\bigr) , e\bigr\rangle \bigr] \\ &=\bigl[1-\sin\bigl(\bar{\theta}^{k}\bigr)\bigr]\mu^{k} = \phi^{k+1}\mu^{0}. \end{aligned}$$

This completes the proof. □

From Remark 1, we have $\phi^{k}=\frac{\|r_{p}^{k}\|_{F}}{\|r_{p}^{0}\|_{F}} =\frac{\|r_{d}^{k}\|_{F}}{\|r_{d}^{0}\|_{F}}$, which implies $\phi^{k}$ represents the relative infeasibility at $(x^{k},y^{k},s^{k})$. Meanwhile, we also have $\phi^{k}=\frac{\mu^{k}}{\mu^{0}}$, which is also the rate of decline of the duality gap *μ*. Thus, if $\phi^{k}\leq\varepsilon$, then Algorithm 1 will stop and we obtain an approximate optimal solution of SO.

#### Remark 2

For Algorithm 1, we choose a particular starting point, which is studied by Zhang [14, 31] and Rangarajan [6]. In what follows, we give the particular starting point.

Let $\check{u}^{0}$ and $(\check{r}^{0}, \check{v}^{0})$ be the minimum-norm solutions to the linear systems $Ax=b$ and $A^{*}y+s=c$, that is,

22 $$ \check{u}^{0}=\arg\min\bigl\{ \|\check{u}\|_{F}:A \check{u}=b\bigr\} ,\qquad \bigl(\check{r}^{0}, \check{v}^{0}\bigr)=\arg \min\bigl\{ \|\check{v}\|_{F}:A^{*}\check{r}+\check{v}=c\bigr\} . $$

Let $\rho^{0}\geq\max\{\|\check{u}^{0}\|_{2},\|\check{v}^{0}\|_{2}\}$ and choose $(x^{0},y^{0},s^{0})$ such that

23 $$ x^{0}=s^{0}=\rho^{0}e, $$

which implies that $x^{0}\in\operatorname{int }\mathcal{K}$, $s^{0}\in \operatorname{int }\mathcal{K} $, $x^{0}-\check{u}^{0}\in \mathcal{K}$, $s^{0}-\check{v}^{0}\in\mathcal{K}$.

Let $\rho^{*}=\min \{\max\{\|x^{*}\|_{2},\|s^{*}\|_{2}\}: x^{*}\in\mathcal {P}^{*},(y^{*},s^{*})\in\mathcal{D}^{*} \}$. In addition, we assume that for some constant $\Psi>0$, it has $\rho^{0}\geq\rho^{*}/\Psi$ (note that we can always increase $\rho^{0}$).

## Complexity analysis

For simplicity, we will often write *x̃*, *y*, *s̃*, *x̄*, *s̄*, *x̂*, *ŝ*, *θ̄* and *ϕ* for $\tilde {x}^{k}$, $y^{k}$, $\tilde{s}^{k}$, $\bar{x}^{k}$, $\bar{s}^{k}$, $\hat{x}^{k}, \hat{s}^{k}$, $\bar{\theta}^{k}$ and $\phi^{k}$, respectively. Moreover, since the NT-scaling point is used in this paper, we can obtain the following special results:

24 $$\begin{aligned}& v:=Q_{p}x=Q_{p^{-1}}s \quad\Leftrightarrow \quad v=\tilde{x}=\tilde{s} \quad\Rightarrow \quad v^{2}=\tilde{x}\circ\tilde{s}=\tilde{w}=Q_{\tilde{x}^{1/2}} \tilde {s}, \end{aligned}$$

25 $$\begin{aligned}& \hat{v}:=Q_{p_{1}}\bar{x}=Q_{p^{-1}_{1}}\bar{s}\quad \Leftrightarrow\quad\hat{v}= \hat {x}=\hat{s} \quad\Rightarrow\quad\hat{v}^{2}=\hat{x}\circ\hat{s}= \hat{w}=Q_{\hat {x}^{1/2}}\hat{s}. \end{aligned}$$

In what follows, we give some fundamental lemmas. Firstly, by the proof procedure of Lemma 4.1 and Lemma 2 in [6, 7], we have the following lemma.

### Lemma 4.1

*Let* $\tilde{x}\in\operatorname{int} \mathcal{K}$, $\tilde{s}\in \operatorname{int} \mathcal{K}$, *x̃*, *s̃* *operator commute*, *then*

(i) *For* $\tilde{q}\in\mathcal{K}$, *then* $\|Q_{\tilde {x}^{1/2}}\tilde{q}\|_{F}\leq\langle\tilde{x},\tilde{q} \rangle$;
(ii) $\lambda_{\max}((L_{\tilde{x}}L_{\tilde{s}})^{-1})\leq 1/\lambda_{\min}(\tilde{w})$,

*where* $\tilde{w}=Q_{\tilde{x}^{1/2}}\tilde{s}$.

### Lemma 4.2

([6, Lemma 2.9])

*For* $x,y\in\mathcal{J}$, *then* $\|x\circ y\|_{F}\leq\|x\|_{F}\|y\|_{F}$.

### Lemma 4.3

([39, Lemma 2.15])

*If* $x\circ s\in\operatorname{int} \mathcal{K}$, *then* $\det(x)\neq0$.

### Lemma 4.4

([5, Lemma 30])

*Let* $(x, s)\in\operatorname{int} \mathcal{K}\times\operatorname{int} \mathcal{K}$, $w=Q_{x^{1/2}}s$, *then we have* $\|w-\mu e\|_{F}\leq\|x\circ s-\mu e\|_{F}$, *and with equality holding if* *x* *and* *s* *operator commute*.

### Technical results

In order to achieve the iteration complexity bounds for the proposed Algorithm 1, we need some technical results.

#### Lemma 4.5

*Let* $(\tilde{x},\tilde{s})\in\mathcal{N}_{F}(\gamma)$ *and* $(\dot{\tilde {x}},\dot{\tilde{s}})$ *be the solution of* (10). *Then*

$$\big\| (\dot{\tilde{x}},\dot{\tilde{s}})\big\| _{2}\leq\sqrt{\mu r}+(1+\sqrt {2})\zeta, $$

*where* $\zeta:=\min\{\|(\check{u},\check{v})\|_{2}:\tilde{A}\check {u}=\tilde{r}_{p},\tilde{A}^{*}\check{r}+ \check{v}=\tilde{r}_{d}\}$, $\| (\check{u},\check{v})\|_{2}:=\sqrt{\|\check{u}\|_{F}^{2}+\|\check{v}\|_{F}^{2}}$.

#### Proof

Let $(\check{u},\check{r},\check{v})\in\mathcal{J}\times {\mathbb {R}^{m}}\times\mathcal{J}$ satisfy equations $\tilde{A}\check{u}=\tilde{r}_{p}$ and $\tilde{A}^{*}\check{r}+\check{v}=\tilde{r}_{d}$. Using system (7) and $v=\tilde{x}=\tilde{s}$, we have

$$\begin{gathered} \tilde{A}(\dot{\tilde{x}}-\check{u})=0, \\ \tilde{A}^{*}( \dot{y}-\check{r})+(\dot{\tilde{s}}-\check{v})=0, \\ L_{v}(\dot{\tilde{x}}-\check{u})+L_{v}(\dot{\tilde{s}}- \check{v})=v^{2} -(L_{v}\check{u}+L_{v} \check{v}). \end{gathered}$$

Multiplying the last equation by $L_{v}^{-1}$, we obtain

26 $$ (\dot{\tilde{x}}-\check{u})+(\dot{\tilde{s}}-\check{v})=v -( \check{u}+\check{v}). $$

Using the definition of $\|(\check{u},\check{v})\|_{2}:=\sqrt{\|\check {u}\|_{F}^{2}+\|\check{v}\|_{F}^{2}}$, we have

27 $$ \|\check{u}\|_{F}+\|\check{v}\|_{F}\leq \sqrt{2}\big\| (\check{u},\check{v})\big\| _{2}. $$

Using (26), (27) and the fact $\langle\dot{\tilde{x}}-\check{u},\dot{\tilde {s}}-\check{v}\rangle=0$, we have

$$\begin{aligned} \big\| (\dot{\tilde{x}},\dot{\tilde{s}})\big\| _{2}&\leq\big\| (\dot{\tilde{x}}- \check {u},\dot{\tilde{s}}-\check{v})\big\| _{2} +\big\| (\check{u},\check{v}) \big\| _{2} \\ &\leq\big\| v-(\check{u}+\check{v})\big\| _{F}+\big\| (\check{u},\check{v}) \big\| _{2} \\ &\leq\|v\|_{F}+\|\check{u}\|_{F}+\|\check{v} \|_{F}+\big\| (\check{u},\check{v})\big\| _{2} \\ &\leq\sqrt{\mu r}+(1+\sqrt{2})\big\| (\check{u},\check{v})\big\| _{2} \\ &=\sqrt{\mu r}+(1+\sqrt{2})\zeta, \end{aligned}$$

where the last inequality uses the result

$$\begin{aligned} \|v\|_{F}=\|\sqrt{\tilde{x}\circ\tilde{s}}\|_{F}\leq\sqrt{ \|\tilde{x}\circ \tilde{s} \|_{F}}\leq\sqrt{\langle\tilde{x}, \tilde{s} \rangle} \leq\sqrt{\mu r}. \end{aligned}$$

The proof is completed. □

Using Remark 2 and the proof techniques of Lemma A.1 in [40], we have the following lemma, which gives the upper bound on *ζ*.

#### Lemma 4.6

*Let* $(\check{u}^{0},\check{r}^{0},\check{v}^{0})$, $(x^{0},y^{0},s^{0})$ *satisfy* (22), (23) *and* $(\check{u},\check{r},\check{v})$ *satisfy the conditions in Lemma* 4.5, *then* $\zeta\leq(5+4\Psi)r\sqrt{\mu}/\sqrt{\beta}$.

#### Lemma 4.7

*Let* $(\tilde{x},\tilde{s})\in\mathcal{N}_{F}(\gamma)$, $\beta=1-\gamma$, *then*

$$\|\dot{\tilde{x}}\|_{F}\|\dot{\tilde{s}}\|_{F}\leq \frac{1}{2}\omega^{2} \mu r^{2}, $$

*where* $\omega=1+(1+\sqrt{2})(5+4\Psi)/\sqrt{\beta}\geq11$.

#### Proof

Using Lemmas 4.5 and 4.6, we have

$$\begin{aligned} \|\dot{\tilde{x}}\|_{F}\|\dot{\tilde{s}}\|_{F}&\leq \frac{1}{2} \bigl[\|\dot {\tilde{x}}\|_{F}^{2}+ \| \dot{\tilde{s}}\|_{F}^{2} \bigr] =\frac{1}{2}\big\| (\dot{ \tilde{x}},\dot{\tilde{s}})\big\| _{2}^{2} \\ &\leq\frac{1}{2} \bigl[\sqrt{\mu r}+(1+\sqrt{2})\zeta \bigr]^{2} \\ &\leq\frac{1}{2} \bigl[\sqrt{\mu r}+(1+\sqrt{2}) (5+4\Psi)r\sqrt{\mu }/ \sqrt{\beta} \bigr]^{2} \\ &\leq\frac{1}{2} \bigl[\sqrt{1/ r}+(1+\sqrt{2}) (5+4\Psi)/\sqrt{\beta } \bigr]^{2}\mu r^{2} \leq\frac{1}{2}\omega^{2} \mu r^{2}, \end{aligned}$$

which completes the proof. □

#### Lemma 4.8

*Let* $(\tilde{x},\tilde{s})\in\mathcal{N}_{F}(\gamma)$, $\tilde{\omega }=Q_{\tilde{x}^{1/2}}\tilde{s}$, $\beta=1-\gamma$, *then*

(i) $\|(\ddot{\tilde{x}},\ddot{\tilde{s}})\|_{2}^{2}\leq\omega^{4} \mu r^{4}/\beta$,
(ii) $\|\ddot{\tilde{x}}\|_{F}\|\ddot{\tilde{s}}\|_{F}\leq\omega^{4} \mu r^{4}/(2\beta)$.

#### Proof

Multiplying the equation of (11) by $L_{v}^{-1}$ and taking norm-squared on both sides, we have

28 $$ \begin{aligned}[b] \|\ddot{\tilde{x}}+\ddot{\tilde{s}}\|_{F}^{2}&= \big\| L_{v}^{-1} (-2\ddot {\tilde{x}} \circ\ddot{\tilde{s}} ) \big\| _{F}^{2}=\big\| (L_{\tilde {x}}L_{\tilde{s}})^{-1/2} (-2\ddot{\tilde{x}} \circ\ddot{\tilde {s}} )\big\| _{F}^{2} \\ &\leq\lambda_{\max}\bigl((L_{\tilde{x}}L_{\tilde{s}})^{-1} \bigr)\|2\ddot{\tilde {x}} \circ\ddot{\tilde{s}}\|_{F}^{2} \leq\frac{4}{\lambda_{\min}({\tilde {\omega}})}\|\ddot{\tilde{x}} \circ\ddot{\tilde{s}} \|_{F}^{2} \\ &\leq\frac{4}{\beta\mu} \bigl[\|\ddot{\tilde{x}}\|_{F} \|\ddot{\tilde{s}} \| _{F} \bigr]^{2} \leq\frac{4}{\beta\mu} \biggl[ \frac{1}{2} \omega^{2} \mu r^{2} \biggr]^{2} =\frac{1}{\beta}\omega^{4} \mu r^{4},\end{aligned} $$

where the second equality uses (24), the first two inequalities follow from Lemma 4.1, the last two inequalities are due to Lemmas 4.2 and 4.7.

Using the fact $\langle\ddot{\tilde{x}},\ddot{\tilde{s}}\rangle=0$ and (28), we have

$$\begin{aligned} \|\ddot{\tilde{x}}\|_{F}\|\ddot{\tilde{s}}\|_{F}\leq \frac{1}{2} \bigl[ \| \ddot{\tilde{x}}\|^{2}+\|\ddot{\tilde{s}} \|_{F}^{2} \bigr] =\frac{1}{2}\|\ddot{\tilde{x}}+ \ddot{\tilde{s}}\|_{F}^{2} \leq\frac{1}{2\beta} \omega^{4} \mu r^{4}. \end{aligned}$$

Therefore, the proof of the lemma is completed. □

The next result follows from Lemmas 4.7 and 4.8.

#### Lemma 4.9

*Let* $(\tilde{x},\tilde{s})\in\mathcal{N}_{F}(\gamma)$, $\beta=1-\gamma$, *then*

- $\|\dot{\tilde{x}}\|_{F}\|\ddot{\tilde{s}}\|_{F}\leq\omega^{3} \mu r^{3}/\sqrt{\beta}$,
- $\|\dot{\tilde{s}}\|_{F}\|\ddot{\tilde{x}}\|_{F}\leq\omega^{3} \mu r^{3}/\sqrt{\beta}$.

### The lower bounds on *θ̄*

In this subsection, we will find a lower bounds of *θ̄* to satisfy (15) and (16). They will play a key role in complexity analysis. Let $\bar{\theta}^{0}=\arg\sin(\frac{\beta\gamma}{2\omega r})$. If we can prove that (15) and (16) hold for all $\theta\in(0,\bar {\theta}^{0}]$, then $\bar{\theta}^{0}$ is one of the lower bounds on *θ̄*. For this purpose, we first give an important lemma.

#### Lemma 4.10

*Let* $(\tilde{x},\tilde{s})\in\mathcal{N}_{F}(\gamma)$, $\beta=1-\gamma$ *and* $\mu(\theta)=(1-\sin(\theta))\mu$ *be defined in* (21), *then*, *for all* $\theta\in(0,\bar{\theta}^{0}]$,

$$\begin{aligned} \big\| \tilde{x}(\theta)\circ\tilde{s}(\theta)-\mu(\theta) e\big\| _{F} \leq 2 \gamma\mu(\theta). \end{aligned}$$

#### Proof

In order to express convenience, we give some notations as follows:

$$\begin{aligned} f_{1}(\theta)&=\bigl(1-\sin(\theta)\bigr) (\tilde{x}\circ\tilde{s}- \mu e),\qquad f_{2}(\theta)= g^{2}(\theta)\dot{\tilde{x}}\circ\dot{ \tilde{s}}, \\ f_{3}(\theta)&= g(\theta)\sin(\theta)\xi, \qquad f_{4}( \theta)= g^{2}(\theta )\ddot{\tilde{x}}\circ\ddot{\tilde{s}}. \end{aligned}$$

Using (14), Lemma 4.2, Lemmas 4.7, 4.8, 4.9, we have

$$\begin{gathered} \big\| f_{1}(\theta)\big\| _{F}\le\bigl(1-\sin(\theta)\bigr)\| \tilde{x}\circ\tilde{s}-\mu e\| _{F}\leq\bigl(1-\sin(\theta)\bigr) \gamma\mu, \\ \big\| f_{2}(\theta)\big\| _{F}= \big\| g^{2}(\theta)\dot{ \tilde{x}}\circ\dot{\tilde{s}}\big\| \leq g^{2}(\theta) \|\dot{ \tilde{x}}\|_{F}\|\dot{\tilde{s}}\|_{F}\leq \frac {1}{2}\sin^{4}(\theta)\omega^{2} \mu r^{2} :=a_{1}, \\ \big\| f_{3}(\theta)\big\| _{F}\leq g(\theta)\sin(\theta)\| \xi \|_{F}\leq\frac {2}{\sqrt{\beta}}\sin^{3}(\theta) \omega^{3} \mu r^{3}:=a_{2}, \\ \big\| f_{4}(\theta)\big\| _{F}\leq g^{2}(\theta)\| \ddot{ \tilde{x}}\circ\ddot{\tilde {s}}\|_{F}\leq\frac{1}{2\beta} \sin^{4}(\theta)\omega^{4} \mu r^{4}:=a_{3}, \end{gathered}$$

where we use $g(\theta)=1-\cos(\theta)\leq\sin^{2}(\theta)$ and the fact

$$\|\xi\|_{F}=\|\dot{\tilde{x}}\circ\ddot{\tilde{s}}+\dot{\tilde{s}} \circ \ddot{\tilde{x}}\|_{F}\leq\|\dot{\tilde{x}}\|_{F}\| \ddot{\tilde{s}}\|_{F}+\| \dot{\tilde{s}}\|_{F}\|\ddot{ \tilde{x}}\|_{F}. $$

In what follows, we will estimate upper bounds on $a_{1}$, $a_{2}$, $a_{3}$ in the interval $(0,\bar{\theta}^{0}]$. Using $\sin(\bar{\theta}^{0})=\frac{\beta \gamma}{2\omega r}$, we have

$$\begin{gathered} a_{1}=\frac{1}{2}\sin^{4}(\theta) \omega^{2} \mu r^{2}\leq\frac{1}{2}\sin ^{4} \bigl(\bar{\theta}^{0}\bigr)\omega^{2} \mu r^{2}\leq \frac{\gamma^{4}\beta^{4}}{2^{5}\omega ^{2} r^{2}}\mu:=b_{1}, \\ a_{2}=\frac{2}{\sqrt{\beta}}\sin^{3}(\theta) \omega^{3} \mu r^{3}\leq \frac {2}{\sqrt{\beta}}\sin^{3} \bigl(\bar{\theta}^{0}\bigr)\omega^{3} \mu r^{3}\leq \frac{\gamma ^{3}\beta^{5/2}}{2^{2}}\mu:=b_{2}, \\ a_{3}=\frac{1}{2\beta}\sin^{4}(\theta) \omega^{4} \mu r^{4}\leq \frac{1}{2\beta }\sin^{4} \bigl(\bar{\theta}^{0}\bigr)\omega^{4} \mu r^{4}\leq \frac{\gamma^{4}\beta ^{3}}{2^{5}}\mu:=b_{3}. \end{gathered}$$

Based on the analysis, we give the desired result, which is

$$\begin{gathered} 2\gamma\mu(\theta)-\big\| \tilde{x}(\theta)\circ\tilde{s}(\theta)-\mu (\theta) e \big\| _{F} \\ \quad\geq 2\gamma\bigl(1-\sin(\theta)\bigr)\mu- \bigl[\big\| f_{1}(\theta) \big\| _{F}+\big\| f_{2}(\theta )\big\| _{F}+\big\| f_{3}( \theta)\big\| _{F}+\big\| f_{4}(\theta)\big\| _{F} \bigr] \\ \quad\geq \gamma\bigl(1-\sin(\theta)\bigr)\mu- [a_{1}+a_{2}+a_{3} ] \\ \quad\geq \gamma\bigl(1-\sin(\theta)\bigr)\mu- [b_{1}+b_{2}+b_{3} ] \\ \quad\geq\gamma\mu \biggl[1-\frac{\gamma\beta}{2\omega r}-\frac{\gamma^{3}\beta ^{4}}{2^{5}\omega^{2} r^{2}}- \frac{\gamma^{2}\beta^{5/2}}{2^{2}}-\frac{\gamma^{3}\beta ^{3}}{2^{5}} \biggr] \\ \quad\geq \gamma\mu \biggl[1-\frac{1}{2^{3}}-\frac{1}{2^{11}}- \frac {1}{2^{6}}-\frac{1}{2^{11}} \biggr]\geq0, \end{gathered}$$

where we use the result of $\tilde{x}(\theta)\circ\tilde{s}(\theta)$ in (13). □

#### Lemma 4.11

*Let* $(\tilde{x},\tilde{s})\in\mathcal{N}_{F}(\gamma)$, *then we have* $\tilde{x}(\theta)\in\operatorname{int} \mathcal{K}$, $\tilde{s}(\theta )\in\operatorname{int} \mathcal{K}$ *for all* $\theta\in(0,\bar{\theta}^{0}]$.

#### Proof

Using Lemma 4.10 and $\gamma\leq1/4$, we have

$$\begin{aligned} \lambda_{\min}\bigl(\tilde{x}(\theta)\circ\tilde{s}(\theta)\bigr)-\mu( \theta)\geq -2\gamma\mu(\theta), \end{aligned}$$

which is equivalent to

29 $$ \lambda_{\min}\bigl(\tilde{x}(\theta)\circ\tilde{s}( \theta)\bigr)\geq(1-2\gamma ) \mu(\theta)\geq0, $$

which furthermore implies $\tilde{x}(\theta)\circ\tilde{s}(\theta)\in \operatorname{int} \mathcal{K}$.

From Lemma 4.3, we have $\det(\tilde {x}(\theta))\neq0$ and $\det(\tilde{s}(\theta))\neq0$. Furthermore, since $\tilde{x}\in\operatorname{int}\mathcal{K}$, $\tilde{s}\in \operatorname{int}\mathcal{K}$, by the continuity, it follows that both $\tilde{x}(\theta)\in\operatorname{int}\mathcal{K}$ and $\tilde {s}(\theta)\in\operatorname{int}\mathcal{K}$ in $[0,\bar{\theta}^{0}]$. The proof is completed. □

From the above analysis, we obtain the result that $\bar{\theta}^{0}$ is one of the lower bounds on *θ̄*.

### Corrector step and iteration complexity

It is well known that an important requirement for the MTY-PC algorithm is that the new iteration point must stay in the given neighborhood, which is equivalent to proving $(\hat{x}({\theta}),\hat{s}({\theta}))\in\mathcal{N}_{F}(\gamma)$. In what follows, we will complete this task.

Using $(\bar{x},\bar{s})=(Q_{p^{-1}}\tilde{x}(\bar{\theta}), Q_{p}\tilde {s}(\bar{\theta}))$ and $(\hat{x},\hat{s})=(Q_{p_{1}}\bar {x},Q_{p_{1}^{-1}}\bar{s})$, we have

$$(\hat{x},\hat{s})=(Q_{p_{1}}\bar{x},Q_{p_{1}^{-1}}\bar {s})= \bigl(Q_{p_{1}}Q_{p^{-1}}\tilde{x}(\bar{\theta}),Q_{p_{1}^{-1}}Q_{p} \tilde {s}(\bar{\theta})\bigr), $$

which implies $(\hat{x},\hat{s})$ in interval $(0,\bar{\theta}^{0}]$ to satisfy the condition in Lemma 4.10, Lemma 4.11. Thus, by Lemma 4.10, Lemma 4.11, we have

30 $$ \big\| \hat{x}\circ\hat{s}-\mu(\theta) e\big\| _{F} \leq2\gamma \mu(\theta), \quad\hat {x}\in\operatorname{int} \mathcal{K}, \hat{s}\in \operatorname{int} \mathcal{K}. $$

#### Lemma 4.12

*Let* $(\triangle\hat{x},\triangle\hat{s})$ *be the solution of* (18), *then we have*

$$\|\triangle\hat{x}\|_{F}\|\triangle\hat{s}\|_{F}\leq \frac{2\gamma ^{2}}{(1-2\gamma)}\mu(\theta) $$

*for all* $\theta\in(0,\bar{\theta}^{0}]$.

#### Proof

Multiplying the last equation in (18) by $L_{\hat{v}}^{-1}$, we obtain

$$\begin{aligned} \triangle\hat{x}+\triangle\hat{s}=L_{\hat{v}}^{-1} \hat{r}_{c}. \end{aligned}$$

Taking norm-squared on both sides of the above equation, we have

$$\begin{aligned} \|\triangle\hat{x}+\triangle\hat{s}\|_{F}^{2}&= \big\| L_{\hat{v}}^{-1}\hat{r}_{c}\big\| _{F}^{2}= \big\| (L_{\hat{x}}L_{\hat{s}})^{-1/2}\hat{r}_{c} \big\| _{F}^{2} \\ &\leq\frac{1}{\lambda_{\mathrm{min}}((L_{\hat{x}}L_{\hat{s}})^{-1})}\|r_{c}\|_{F}^{2} \leq\frac{1}{\lambda_{\mathrm{min}}(\hat{w})}\big\| \mu(\theta) e-\hat{x}\circ\hat {s}\big\| _{F}^{2} \\ &\leq\frac{1}{(1-2\gamma)\mu(\theta)}\bigl(2\gamma\mu(\theta)\bigr)^{2} =\frac{4\gamma^{2}}{(1-2\gamma)}\mu(\theta), \end{aligned}$$

where $\hat{r}_{c}=(1-\sin(\bar{\theta}))\mu e-\hat{x}\circ\hat{s}$, the second equality uses (25), the second inequality follows from Lemma 4.1, the third inequality is due to (29), (30).

Using the conclusion as above and the fact $\langle\triangle\hat{x}, \triangle\hat{s}\rangle=0$, we have

$$\begin{aligned} \|\triangle\hat{x}\|_{F}\|\triangle\hat{s}\|_{F}&\leq \frac{1}{2} \bigl[\| \triangle\hat{x}\|_{F}^{2}+\| \triangle\hat{s}\|_{F}^{2} \bigr] =\frac{1}{2}\| \triangle\hat{x}+\triangle\hat{s}\|_{F}^{2}\leq \frac{2\gamma ^{2}}{(1-2\gamma)}\mu(\theta), \end{aligned}$$

which completes the proof. □

#### Lemma 4.13

*Let* $(\hat{x}({\theta}),\hat{s}({\theta}))$ *be defined in* (19), $\theta\in(0,\bar{\theta}^{0}]$, *then we have*

$$\bigl(\hat{x}({\theta}),\hat{s}({\theta})\bigr)\in\mathcal{N}_{F}( \gamma). $$

#### Proof

Using Lemma 4.4, (20) and Lemma 4.12, we have

$$\begin{aligned} \big\| \hat{w}(\theta)-\mu(\theta) e\big\| _{F} &\leq\big\| \hat{x}(\theta)\circ\hat {s}(\theta)-\mu(\theta) e\big\| _{F} \\ &=\|\triangle\hat{x}\circ\triangle\hat{s}\|_{F}\leq\|\triangle\hat{x} \| _{F} \|\triangle\hat{s}\|_{F} \\ &\leq\frac{2\gamma^{2}}{(1-2\gamma)}\mu(\theta)\leq\frac{2\gamma }{1-2\gamma}\gamma\mu(\theta) \leq\gamma\mu(\theta), \end{aligned}$$

where $\hat{w}(\theta)=Q_{\hat{x}(\theta)^{1/2}}\hat{s}(\theta)$, the last inequality follows from $\gamma\leq1/4$.

Using the proof technique that is similar to Lemma 4.11, we have

$$\hat{x}(\theta)\in\operatorname{int} \mathcal{K},\qquad \hat{s}(\theta)\in \operatorname{int} \mathcal{K}. $$

Taking into account the above factors, we have $(\hat{x}({\theta}),\hat {s}({\theta}))\in\mathcal{N}_{F}(\gamma)$. □

The following theorem gives an upper bound for the number of iterations in which Algorithm 1 stops with an *ε*-approximate solution.

#### Theorem 4.14

*Let* $r_{p}^{k}$, $r_{d}^{k}$, $\mu^{k}$ *be defined in Remark* 1, *then Algorithm* 1 *will terminate in* $\mathcal{O}(r\log \varepsilon^{-1})$ *iterations such that*

$$\big\| r_{p}^{k}\big\| \leq\varepsilon\big\| r_{p}^{0} \big\| ,\qquad \big\| r_{d}^{k}\big\| \leq\varepsilon\big\| r_{d}^{0} \big\| , \qquad\mu^{k}\leq\varepsilon\mu^{0}. $$

#### Proof

By using $\bar{\theta}^{0}=\arg\sin(\frac{\beta\gamma}{2\omega r})\leq \bar{\theta}$, we have

$$\begin{aligned} \phi^{k}=\prod^{k-1}_{i=0} \bigl(1-\sin\bigl(\bar{\theta}^{i}\bigr)\bigr)\leq\prod ^{k-1}_{i=0}\bigl(1-\sin\bigl(\bar{ \theta}^{0}\bigr)\bigr)\leq\bigl(1-\sin\bigl(\bar{\theta}^{0} \bigr)\bigr)^{k}\leq \varepsilon, \end{aligned}$$

which implies

$$\begin{aligned} k\geq\frac{1}{\sin(\bar{\theta}^{0})}\log\varepsilon^{-1}\geq \frac {2\omega}{\beta\gamma}r \log\varepsilon^{-1}, \end{aligned}$$

where we use the identity $\log(1+t)\leq t$ for all $t>-1$.

Therefore, Algorithm 1 terminates after at most $\mathcal {O}(r\log\varepsilon^{-1})$ steps. Meanwhile, by using Remark 1, we have

$$\begin{aligned} \phi^{k}=\frac{\|r_{p}^{k}\|_{F}}{\|r_{p}^{0}\|_{F}} =\frac{\|r_{d}^{k}\|_{F}}{\|r_{d}^{0}\|_{F}}=\frac{\mu^{k}}{\mu^{0}}\leq \varepsilon. \end{aligned}$$

This completes the proof. □

## Conclusion

For the SO problem, we have proposed an MTY-PC infeasible-IPM, which requires two matrix factorizations and at most three back-solves for each iteration. In order to improve the iteration complexity, we adopt the arc-search strategy that was proposed by Yang [32–34]. Moreover, the proposed algorithm can ensure that the duality gap and the infeasibility have the same rate of decline. Finally, by applying the EJA tool to our analysis, we established the iteration complexity $\mathcal{O}(r\log\varepsilon^{-1})$ for the NT-direction.
